# Cardiac autonomic activity and blood pressure among Nunavik Inuit adults exposed to environmental mercury: a cross-sectional study

**DOI:** 10.1186/1476-069X-7-29

**Published:** 2008-06-06

**Authors:** Beatriz Valera, Eric Dewailly, Paul Poirier

**Affiliations:** 1 Public Health Research Unit, 2875 Boulevard Laurier, Édifice Delta 2, bureau 600, G1V 2M2, Quebec (Qc), Canada; 2 Quebec Heart and Lung Institute, Laval Hospital Research Centre, 2725 Chemin Sainte-Foy, G1V 4G5, Québec (Qc), Canada; 3 Faculty of Pharmacy, Laval University, Quebec (Qc), Canada

## Abstract

**Background:**

Mercury is a contaminant that reaches high levels in Nunavik (North of Quebec). It is transformed into methylmercury (MeHg) and accumulated in marine mammals and predator fish, an important part of the traditional Inuit diet. MeHg has been suggested to affect BP in adults and children while the influence on HRV has only been studied in children. We aimed to assess the impact of MeHg levels on HRV and BP in Inuit adults from Nunavik.

**Methods:**

In the fall of 2004, the «Qanuippitaa?» Health Survey was conducted in Nunavik (Quebec, Canada) and information on HRV was collected among 280 adults aged 40 years and older. Indicators of the time and frequency domains of HRV were derived from a 2-hour Holter recording. BP was measured according to the Canadian Coalition for High Blood Pressure technique. Pulse pressure (PP) was the difference between systolic (SBP) and diastolic blood pressure (DBP). Blood mercury concentration was used as exposure biomarker. Statistical analysis was conducted through linear regression and multivariable linear regression was used to control for confounders.

**Results:**

Mercury was negatively correlated with low frequency (LF) (r = -0.18; p = 0.02), the standard deviation of RR intervals (SDNN) (r = -0.14; p = 0.047) and the coefficient of variation of RR intervals (CVRR) (r = -0.18; p = 0.011) while correlations with other HRV parameters did not reach statistical significance. After adjusting for confounders, the association with LF (beta = -0.006; p = 0.93) became non significant. However, the association with SDANN became statistically significant (beta = -0.086; p = 0.026) and CVRR tended to decrease with blood mercury concentrations (beta = -0.057; p = 0.056). Mercury was positively correlated with SBP (r = 0.25; p < 0.0001) and PP (r = 0.33; p < 0.0001). After adjusting for confounders, these associations remained statistically significant (beta SBP = 4.77; p = 0.01 and beta PP = 3.40; p = 0.0036). Moreover, most of the HRV parameters correlated well with BP although SBP the best before adjustment for mercury exposure.

**Conclusion:**

The results of this study suggest a deleterious impact of mercury on BP and HRV in adults. SBP and PP increased with blood mercury concentrations while SDANN decreased with blood mercury concentrations.

## Background

Mercury is a contaminant of great concern in the Arctic and in northern Quebec (Nunavik) [1]. Mercury can be transformed into methylmercury (MeHg) by biomethylation of the inorganic mercury present in aquatic sediments. It accumulates in the aquatic food chain and reaches its highest concentrations in long-lived and predatory fish and whales [2,3]. Arctic populations are highly exposed to MeHg as their traditional diet is still largely based on fish and marine mammal consumption.

A study carried out between 1977 and 1982 including 142 adults of Nunavik (Northern Quebec, Canada) revealed a mean mercury blood concentration of 240 nmol/L [4]. Ten years later, another study was conducted by Dewailly *et al*. [5] and the investigators noticed that blood mercury levels had decreased (mean = 109 nmol/L) but still remained high compared to populations in Southern Quebec (mean = 3.7 nmol/L) [6] or Nunavik residents of Caucasian origin (mean = 18.6 nmol/L) [5]. They also reported that Inuit in the 45 to 75-year age group had the highest mean blood mercury concentrations.

MeHg is well known for its toxic effect on the central nervous system (CNS) but recently, some authors have suggested that it can also interfere with the normal function of the cardiovascular system [7-11]. Some studies have reported an association between mercury and myocardial infarction [7], high blood pressure, [8,9] and reduced heart rate variability (HRV) [8,10-12]. This latter reflects the cardiac parasympathetic and sympathetic activities of the autonomic nervous system (ANS). Reduced HRV can cause ventricular fibrillation, which can lead to sudden cardiac death [13-17]. A decrease in HRV has also been associated with other cardiac events (angina pectoris, myocardial infarction, coronary heart disease death, or congestive heart failure) [18] and all causes of mortality [19].

The impact of MeHg on BP has been reported in children [8,11] and adults [9,20] while the influence of MeHg on HRV has only been reported in subjects exposed *in utero *[8,10-12]. In a cohort study of 7-year old children from the Faroe Islands, the coefficient of variation of heart rate (CVRR) in boys decreased by 47% in a range of cord blood concentrations from 1 to 10 μg/L (5–50 nmol/L). This was depicted as an indication of parasympathetic nervous dysfunction in children exposed to low doses of MeHg during the prenatal period [8]. This cohort was examined at 14 years of age and a 2.7% decrease in the heart rate coefficient of variation was detected. Moreover, a decrease of 6.7% in the parasympathetic and sympathetic activity was reported [11]. Oka *et al*. [10] conducted a case-control study to assess the chronic effect of exposure to high doses of MeHg on HRV in subjects suffering from fetal Minamata disease (FMD). HRV indices such as the R-R interval (NN) and high frequency (HF) were lower in FMD patients. Furthermore, the standard deviation of R-R intervals (SDNN) and CVRR tended to be lower in the FMD group but this difference did not reach statistical significance. More recently, Murata *et al. *[12] reported an association between MeHg exposure at parturition and decreased vagal modulation of cardiac autonomic function in Japanese children aged 7 years. Regarding BP, a deleterious impact of prenatal MeHg exposure on SBP was observed in children from Faroe Island at 7 years of age [8]. In adults, MeHg has been associated with decreasing DBP and increasing PP in Greenlanders [9] and increased risk of hypertension in inhabitants of the Brazilian Amazon [20].

Taking into account that the impact of background mercury levels on HRV in adults environmentally exposed has never been reported and that results on mercury and BP are inconsistent, we aimed to assess the impact of blood mercury concentrations on HRV and BP in Nunavik Inuit adults taking into account the influence of n-3 fatty acids and other possible confounding factors. We hypothesized that increasing blood mercury levels were associated with reduced HRV and increased BP.

## Methods

In the fall of 2004, the «Qanuippitaa?» Health Survey was conducted in the 14 coastal communities of Nunavik. The target population included all permanent residents, except for non-Inuit households and individuals living full time in public institutions. The survey plan was a complex two-stage stratified random sampling. The first stage was to select a stratified random sample of private Inuit households with proportional allocation. The community was the only stratification variable used. This stratification allowed the representation of the target population to be up to standard. Since home addresses (civic numbers) in some municipalities are consecutive, the survey frame was sorted first by home addresses, followed by a systematic draw of a predetermined number of households to avoid selection of two immediate neighbours. Since many Inuit regularly move from one house to another, it was decided to sample households instead of individuals. The assumption was that recruiting a member of a household rather than a specific individual, would increase coverage of the target population. To obtain a good representation of each community, a proportional allocation of sample units corresponding to the size of each village was chosen. It was important to choose households from all 14 communities since the distances separating villages could be associated with significant differences in lifestyle. In the second stage, all eligible people were asked to participate according to the survey steps or instruments. Among the 677 households visited by the interviewers, 521 agreed to participate to the survey. In the present study, we used the 2-hour Holter data collected only among 280 adults 40 years and older.

All the information used in this study was gathered on board the research icebreaker «Amundsen». For this purpose, each participant was invited to fill out questionnaires and to attend a clinical evaluation. Each individual who accepted to participate in the survey signed a consent form. The study protocol was approved by the ethics committee of Laval University. Questionnaires were used to gather information regarding age, gender, smoking habits, alcohol consumption, total income in the last 12 months as well as leisure time physical activity. Leisure time physical activity was estimated using the energy expenditure during exercise [MET (kcal/kg/week) = duration*frequency*intensity]. During the clinical session, blood samples were collected and anthropometric and physiological measurements were taken. As anthropometric variable, we measured waist circumference (WC), which was obtained using a graduated tape when subjects were in a standing position [21].

HRV indices were derived from a 2-hour Holter monitoring system (GE MARQUETTE SERIE 8500) with a recording frequency of 128 Hz. Seven leads (derivations V5, V1, and AVF) were installed when subjects arrived to the clinic after blood sample was taken. During 2 hours, subjects stayed at the clinic and completed questionnaires and had anthropometrics measurements. Interpretation and extraction of HRV parameters were performed automatically by using the software provided by General Electric (MARS PC Ambulatory ECG Analysis System) while complete signal was carefully edited using visual checks and manual corrections of individual RR intervals and QRS complex classifications. For the calculation of the RR parameters, only R-R intervals between QRS complexes of sinusal origin were used. Intervals whose duration was < 80% or > 120% of that of the running R-R average were excluded. Time domain parameters included the average of all R-R intervals (NN), standard deviation of R-R intervals (SDNN), standard deviation of the average R-R intervals calculated over 5-minute periods (SDANN), the square root of the mean squared differences of successive R-R intervals (rMSSD) and the proportion of interval differences of successive R-R intervals > 50 ms (pNN50). rMSSD and pNN50 are indices of cardiac parasympathetic modulation. CVRR was calculated as (SDNN/NN) × 100. Fast Fourier transformation was used to compute frequency domain measurements. Thus, it was possible to calculate very low frequency (VLF = 0.0033–0.04 Hz), which may be an index of parasympathetic activity and neuroendocrine and thermogenesis stimulus, low frequency (LF = 0.04–0.15 Hz) which represents both sympathetic and parasympathetic activity, and high frequency (HF = 0.15–0.40 Hz) which is an index of solely parasympathetic activity. The LF/HF ratio represents the sympatho-vagal balance [22,23]. BP was measured according to the Canadian Coalition for High Blood Pressure prevention and control using mercury sphygmomanometers, 15-inch stethoscopes, and cuffs sized to the subjects' arms [24]. Prior to having their BP taken, subjects had to have rested for five minutes and not eaten or smoked for at least thirty minutes. Each subject had two BP readings and means of systolic blood pressure (SBP) and diastolic blood pressure (DBP) were calculated. Pulse pressure (PP) was calculated as the difference between SBP and DBP.

Blood mercury and selenium concentrations were measured by inductively coupled plasma mass spectrometry (ICP-MS). For mercury determination, blood samples were diluted 20 fold in a solution containing ammonium hydroxide before analysis. Concentrations of plasma total cholesterol (total-C), triglycerides, low-density lipoprotein cholesterol (LDL-cholesterol) and high-density lipoprotein cholesterol (HDL-cholesterol) were analysed according to methods of the Lipid Research Clinics (US Department of Health). Cholesterol and triglycerides concentrations were determined in plasma and in lipoprotein fractions using an Auto-Analyzer II (Technicon Instruments Corporation, Tarrytown, New York). The HDL fraction was obtained after precipitation of LDL in the infranatant with heparin and manganese chloride. Low-density lipoprotein (LDL-cholesterol) was calculated using Friedewald's formula [25]. Insulin determination was performed with an Auto-analyzer Roche Modular analytics E170 (Elecsys module) using a commercial double-antibody radioimmunoassay. Plasma glucose was measured enzymatically through a reaction with hexokinase. Insulin sensitivity was estimated from the HOMA model (Homeostasis model assessment) as fasting insulin times fasting glucose/22.5 [26]. The fatty acid composition of the erythrocyte membranes was measured after membrane purification, chloroform/methanol lipid extraction [27] and methylation of fatty acids, followed by capillary gas-liquid chromatography using a DB-23 column (39 × 0.25 mm ID × 0.25 μm thickness) in a Hewlett-Packard GC chromatograph.

### Statistical analyses

As an initial step, descriptive statistics for all variables were produced. Results are presented as arithmetic mean ± standard deviation for continues variables with normal distribution. Log-transformation was applied to variables with skewed distribution and geometric means were calculated. Proportions are presented for categorical data. Taking into account the complex sampling design used in this study, sampling weights were incorporated into the calculation of descriptive statistics as well as linear regression parameters. Weighting participants answers takes into account the probability of selecting each individual as induced by the design of the survey, the rates of non-response and differences observed between the sample and the population. The weight corresponds to the number of persons in the entire population who are represented by the respondent. Thus, the estimates generated by using sampling weights could be generalized to the entire population of Nunavik. Details on methodology are available in the methodological report of the survey [28].

The relationship between blood mercury levels and the outcomes (Holter and BP parameters) was analysed using Pearson's correlation. The influence of potential confounding factors was examined through multivariable regressions. Variables assessed as potential confounders in HRV models were gender, age, waist circumference (WC), insulin sensitivity (HOMA-IR), LDL-cholesterol, HDL-cholesterol, triglycerides, smoking habits, alcohol consumption, physical activity, socio-economic status (measured as total income), n-3 fatty acids [Docosahexanoic acid (DHA) and Eicosapentanoic acid (EPA)]. In BP models, we also considered selenium as potential confounder. All variables were included in the initial models and selected as confounders if their exclusion modulated the regression coefficients by more than 10%. Squared partial correlation coefficients of the variables retained in the final models were also calculated in order to determine which of them explained a significant proportion of the model's variance. Final models were analysed to verify if the assumptions of a linear regression were respected. Thus, linearity, normality and homoscedasticity (homogeneity of variance) of the residuals were examined using graphic plots of the jackknife residuals versus predicted values of the dependent and independent variables. Collinearity between variables included in the final models were also analysed to avoid including in the same model two variables highly correlated. A p < 0.05 was considered of statistical significance. All analyses were performed using SAS v. 9.1 and SUDAAN v.9.3 software (SAS Institute, Cary, NC).

Sensitivity analyses were performed in order to assess if exclusion of individuals with hypertension, myocardial infarction, stroke and other cardiovascular diseases was necessary for HRV analyses. We also verified if exclusion of individuals with antiarrhythmic or beta blocker medication was necessary. For BP analyses, we verified if exclusion of individuals taking medication for hypertension changed the regression coefficients.

Owing to the complex sampling method used in this study, the bootstrap technique was selected to determine variances in the descriptive statistics as well as the regression coefficients derived from the sampling design [28-30]. This technique provides precision measurements for estimates obtained from a complex sample design. The bootstrap is in fact a re-sampling method that consists of drawing subsamples (500 subsamples were used in this study) from the original sample and generating estimates for each of those subsamples. An estimation of the sampling variance is deduced by measuring the dispersion between those estimations. In order to extract an estimate for each subsample that could be inferred to the entire population, sample weighting must be used. It involves the production of a set of weights for each subsample. These sets of weights are called bootstrap weights. The calculation of sampling weights as well as bootstrap weights for this survey was done by the Institut de la Statistique du Québec. Details on methodology is available in the methodological report of the survey [28].

## Results

During the survey, 472 individuals of 40 years and over were eligible to the HRV session whereas 280 (59%) accepted to participate. For the statistical analyses, we excluded 69 individuals due to technical problems related to HRV (artefacts, bad connections, etc), 1 individual with blood mercury data not available and 5 individuals who were not Inuit. Thus, data on HRV and BP were available from 205 individuals (43%).

Characteristics of the participants are presented in Table 1. The mean age of the participants was 52.1 years and the study sample included 85 men (mean age: 53.0 years) and 120 women (mean age: 51.2 years). The arithmetic mean of blood mercury concentration was 133.2 nmol/L (27 μg/L) ranging from 2.4 to 760 nmol/L while geometric mean was 97.8 nmol/L (19.6 μg/L). Mercury blood concentrations increased with age (r = 0.35; p < 0.0001) and were similar in women and men (p = 0.63).

**Table 1:** Characteristics of the participants

**Variables**	**N**	**Arithmetic mean (SD)**	**Geometric mean**	**Range**
Age (years)	205	52.1 (28.3)		40 – 73
Gender (%)	205			
Female		120 (58.6%)		
Male		85 (41.5%)		
Mercury (nmol/L)^§^	205	132.9 (329.8)	97.8	2.4 – 760.0
Fasting insulin (pmol/L)^§^	203	68.9 (204.5)	52.1	13.0 – 448.0
Fasting glucose (mmol/L)^§^	202	4.9 (3.7)	4.8	2.8 – 15.1
HOMA-IR ^§^	201	16.9 (77.3)	11.0	1.9 – 244.3
Total-Cholesterol (mmol/L)	202	5.4 (2.8)		3.3 – 8.2
LDL-Cholesterol (mmol/L)	202	3.1 (2.7)		0.7 – 6.1
HDL-Cholesterol (mmol/L)	202	1.7 (1.6)		0.8 – 4.3
Triglycerides (mmol/L)^§^	202	1.3 (2.4)	1.1	0.3 – 5.5
EPA (% fatty acids)^§^	205	2.5 (4.2)	2.1	0.50 – 7.27
DHA (% fatty acids)	205	6.7 (5.1)		1.56 – 10.09
WC (cm)	204	96.0 (41)		66.0 – 136.0
Smoking habits (%)	198			
Daily smoker		116 (59.0%)		
Occasionally		7 (3.5%)		
Not at all		75 (37.9%)		
Physical activity (kcal/kg/week) ^&^	130	43.9 (186)	20.2	0 – 364
Total income (%)	169			
Less than $20,000		80 (47.3%)		
$20,000 to less than $40,000		46 (27.2%)		
$40,000 to less than $60,000		31 (18.3%)		
$60,000 or more		12 (7.1%)		
Alcohol consumption (%)	177			
Yes		141 (79.7%)		
No		36 (20.3%)		

^§ ^Variables not normally distributed; HOMA-IR: insulin sensitivity; EPA: Eicosapentaenoic acid; DHA: Docosahexaenoic acid; WC: Waist circumference; LDL-cholesterol: Low density lipoprotein; HDL-cholesterol: high density lipoprotein;

^&^Physical activity was calculated as: MET = duration*frequency*intensity of the exercise.

Holter and BP parameters are presented in Table 2. Means of SBP and DBP were 123 mmHg and 75 mmHg respectively. All HRV variables but NN showed a skewed distribution. The correlations between mercury and BP and Holter parameters are presented in Table 3. Blood mercury concentration was negatively correlated with LF (r = -0.18; p = 0.02), SDNN (r = -0.14; p = 0.047) and CVRR (r = -0.18; p = 0.011) while correlations with other HRV indices did not reach statistical significance. Mercury was positively correlated with SBP (r = 0.25; p < 0.0001) and PP (r = 0.33; p < 0.0001).

**Table 2:** Time and frequency domains parameters

**HRV and BP parameters**	**Arithmetic mean (SD)**	**Geometric Mean**	**Range**
VLF (ms^2^)^§^	890 (2108)	690/6.5 ^†^	45 – 5999
LF (ms^2^)^§^	574 (2006)	376/5.9 ^†^	16 – 6596
HF (ms^2^)^§^	206 (726)	134/4.9 ^†^	16 – 2329
LF/HF^§^	3.2 (5.4)	2.8	0.4 – 13
NN (ms)	810 (364)		557 – 1167
SDNN (ms)^§^	80 (84)	76	24 – 226
SDANN (ms)^§^	51 (67)	47	12 – 189
RMSSD (ms)^§^	30 (37)	28	12 – 93
PNN50 (%) ^§^	10 (31)	5	0 – 57
SBP (mm Hg)	123 (37)		85 – 187
DBP (mm Hg)	75 (21)		55 – 111
PP (mm Hg)	48 (27)		23 – 94

^§ ^Variables not normally distributed; ^† ^for VLF, LF and HF, the Ln mean is also shown; SBP: systolic blood pressure, DBP: diastolic blood pressure, PP: pulse pressure.

**Table 3:** Results of simple and multivariable regression between mercury and HRV and BP parameters

**Mercury (nmol/L)**^§^				
**HRV and BP parameters**	**Simple analysis**		**Multivariable analysis**	
	
	**r Pearson**	**Crude β (95% CI)**	**Adjusted β (95% CI)**	**Model R**^2^

VLF (ms^2^)^§ a^	-0.13	-0.12 (-0.26, 0.03)	-0.06 (-0.20, 0.07)	0.12**
LF (ms^2^)^§ b^	-0.18*	-0.20* (-0.38, -0.03)	-0.01 (-0.18, 0.16)	0.21***
HF (ms^2^)^§ c^	-0.11	-0.12 (-0.27, 0.03)	-0.01 (-0.17, 0.15)	0.17**
LF/HF ^§ d^	-0.10	-0.22 (-0.51, 0.07)	0.01 (-0.33, 0.34)	0.24***
NN (ms) ^e^	0.04	6.32 (-13.62, 26.27)	-5.75 (-27.90, 16.41)	0.19***
SDNN (ms)^§ f^	-0.14*	-0.06* (-0.12, -0.001)	-0.056 (-0.12, 0.01)	0.09**
SDANN (ms)^§ g^	-0.09	-0.05 (-0.11, 0.01)	-0.086* (-0.16, -0.01)	0.02
rMSSD (ms) ^§ h^	-0.12	-0.06 (-0.12, 0.01)	-0.03 (-0.09, 0.04)	0.12**
pNN50 (%) ^§ i^	-0.12	-0.19 (-0.39, 0.02)	-0.09 (-0.32, 0.13)	0.12*
CVRR (%) ^§ j^	-0.18*	-0.07* (-0.12, -0.01)	-0.057 (-0.11, 0.001)	0.03*
SBP (mm Hg) ^k^	0.25***	5.20*** (2.70, 7.70)	4.77* (1.12 – 8.42)	0.27***
DBP (mm Hg) ^l^	0.01	0.13 (-1.44, 1.71)	1.99 (-0.29, 4.29)	0.08*
PP (mm Hg) ^m^	0.33***	5.07*** (3.15 – 6.99)	3.40* (1.11 – 5.69)	0.41***

* p < 0.05; ** p < 0.01; *** p < 0.0001; ^§ ^Log-transformed variables; ^a ^model adjusted for HDL, WC and DHA; ^b ^model adjusted for smoking, DHA, triglycerides, EPA, WC, HOMA-IR and age; ^c ^model adjusted for smoking, triglycerides, EPA, alcohol, DHA, HOMA-IR, HDL, gender and WC; ^d ^model adjusted for alcohol, DHA, HDL, EPA, LDL, triglycerides, age, HOMA-IR, WC, gender and socio-economic status; ^e ^model adjusted for total income, EPA, smoking, gender, HOMA-IR, HDL and age; ^f ^model adjusted for EPA and HOMA-IR; ^g ^model adjusted for EPA; ^h ^model adjusted for smoking, HDL, DHA, WC and HOMA-IR; ^i ^model adjusted for smoking, HDL, HOMA-IR, DHA, age and WC; ^j ^model adjusted for EPA; ^k ^model adjusted for age, WC and selenium; ^l ^model adjusted for EPA, selenium and triglycerides; ^m ^model adjusted for selenium, WC and age.

The adjusted regression coefficients are presented in Table 3. The significant association between mercury and LF observed in simple correlation became non significant after adjusting for confounding factors. However, the association with SDANN became statistically significant (beta = -0.086, p = 0.026) after adjusting for confounders. There was a trend for SDNN (beta = -0.056, p = 0.10) and CVRR (beta = -0.057, p = 0.056). Mercury remained positively associated with SBP (beta = 4.77; p = 0.01) and PP (beta = 3.40; p = 0.0036) after adjusting for confounders.

Squared partial correlation coefficients for mercury as well as confounders included in final models are presented in Table 4. Mercury explained 4.14% and 4.76% of the variance in SBP and PP models. WC and age also explained a significant proportion of the variance in SBP and PP models while DBP was explained by triglycerides. In HRV models, insulin sensitivity (evaluated as HOMA-IR) explained a significant proportion of the variance in most models (SDNN, NN, HF, LF) while age explained a significant proportion of the variance only in LF, NN and LF/HF ratio models. The proportion of the variance explained by gender was significant in HF and LF/HF ratio models while HDL explained a significant proportion of the variance in VLF, rMSSD and pNN50 models. Finally, waist circumference explained a significant proportion of the variance in VLF, HF, and rMMSD.

**Table 4:** Squared partial correlation coefficients

**HRV and BP parameters**	**Independent variables**	**Adjusted β**	**Partial R^2 ^(%)**	**p-value**
VLF (ms^2^) ^§^	Mercury ^§^	-0.06	0.43	0.38
	WC	-0.02	10.17	<0.0001
	HDL-cholesterol	-0.34	5.52	0.004
LF (ms^2^)^§^	Mercury ^§^	-0.006	0.003	0.94
	HOMA-IR	-0.22	2.66	0.0178
	Age	-0.03	6.15	0.0069
HF (ms^2^)^§^	Mercury ^§^	-0.01	0.01	0.88
	HOMA-IR^§^	-0.19	1.80	0.0466
	WC	-0.01	2.34	0.0102
	Gender	0.39	4.17	0.0011
LF/HF ^§^	Mercury ^§^	0.01	0.001	0.97
	Age	-0.05	6.32	0.0002
	Gender	-1.19	11.05	<0.0001
NN (ms)	Mercury ^§^	-5.75	0.12	0.61
	Age	4.63	8.66	<0.00010
	HOMA-IR^§^	-37.63	5.01	.0028
SDNN (ms)^§^	Mercury ^§^	-0.056	1.25	0.10
	HOMA-IR^§^	-0.12	7.95	0.0001
SDANN (ms)^§^	Mercury ^§^	-0.086	1.86	0.026
rMSSD (ms)^§^	Mercury ^§^	-0.03	0.29	0.395
	HDL	-0.10	1.66	0.0488
	WC	-0.01	3.69	0.0008
pNN50 (%) ^§^	Mercury ^§^	-0.09	0.28	0.43
	HDL	-0.36	1.88	0.032
	WC	-0.03	3.99	0.0006
CVRR (%) ^§^	Mercury ^§^	-0.057	1.66	0.056
SBP (mm Hg)	Mercury ^§^	4.77	4.14	0.01
	Selenium ^§^	-6.45	2.92	< 0.0001
	WC	0.29	7.03	0.0357
	Age	0.59	11.27	< 0.0001
DBP (mm Hg)	Mercury ^§^	1.99	1.57	0.087
	Triglycerides ^§^	4.76	6.68	< 0.0001
PP (mm Hg)	Mercury ^§^	3.40	4.76	0.0036
	WC	0.12	2.83	0.0083
	Age	0.68	27.92	< 0.0001

In this table, we present only the confounding factors that explain a significant proportion of the variance. ^§ ^Log-transformed variables; HOMA-IR: Homeostasis model assessment; WC: Waist circumference.

From 205 individuals included in the final analysis, 18 had a stroke, 8 had a myocardial infarction, 55 had hypertension and 22 suffered from other cardiovascular diseases. Sensitivity analyses showed that most of the HRV parameters were similar to those from individuals not suffering from cardiovascular diseases. Also, 23 individuals were taking antiarrhythmic or beta blocker medication. These individuals had lower LF, LF/HF ratio and higher NN than individuals without antiarrhythmic or beta blocker medication. Statistical analyses were conducted excluding individuals with antiarrhythmic or beta blocker medication and regression coefficients did not change significantly. For BP analyses, sensitivity analyses showed that exclusion of individuals taking medication for hypertension did not change significantly the regression coefficients. Thus, these individuals were not excluded from the analyses.

In the final models, the analysis of the jackknife residuals showed that assumptions of the linear regression had been respected. Jackknife residuals were normally distributed and the analysis of the homoscedasticity showed a homogeneous variance distribution. Linearity assumption was also respected in all models.

Since mercury affects both BP and HRV, and since both are indicators of cardiovascular disease, we studied the relationship between HRV and BP (Table 5).

**Table 5:** HRV parameters (log-transformed) as predictors of blood pressure

	SBP (mm Hg)		DBP (mm Hg)	
HRV ^§ ^parameters	Pearson r (p-value)	Partial correlation coefficient ^&^(p-value)	Pearson r (p-value)	Partial correlation coefficient ^&^(p-value)

VLF (ms^2^)	-0.14 (0.0227)	-0.12 (0.05)	-0.13 (0.07)	-0.13 (0.07)
LF (ms^2^)	-0.27 (< 0.0001)	-0.25 (0.0002)	-0.16 (0.013)	-0.16 (0.01)

HF (ms^2^)	-0.19 (0.0063)	-0.17 (0.01)	-0.22 (0.0005)	-0.22 (0.0007)
LF/HF	-0.14 (0.018)	-0.11 (0.04)	0.11 (0.08)	0.11 (0.069)

SDNN (ms)	-0.13 (0.036)	-0.11 (0.07)	-0.16 (0.007)	-0.16 (0.007)
SDANN (ms)	-0.07 (0.30)	-0.05 (0.41)	-0.14 (0.019)	-0.14 (0.019)
rMSSD (ms)	-0.12 (0.059)	-0.11 (0.11)	-0.20 (0.002)	-0.20 (0.003)
pNN50 (%)	-0.13 (0.047)	-0.11 (0.11)	-0.17 (0.015)	-0.17 (0.019)
CVRR (%)	-0.24 (< 0.0001)	-0.21 (0.0010)	-0.13 (0.036)	-0.13 (0.036)

^§ ^Log-transformed variables; ^&^partial correlation coefficients adjusted for blood mercury concentrations

Most of the HRV parameters correlated well with BP although SBP the best before adjustment for mercury exposure.

## Discussion

To our knowledge, we reported the first study on HRV conducted in adults exposed to MeHg through environmental background exposure. We observed a diminution of parasympathetic activity with higher blood mercury concentrations. In multivariable analyses, only the association with SDANN was statistically significant. However, the associations with other HRV parameters were in the same direction as those reported in previous studies [11,12] and a trend was observed for SDNN and CVRR. BP was also impacted by mercury in this population and SBP and PP increased with blood mercury concentrations.

Of particular interest, 50% of the participants had mercury concentrations in the range considered at risk according to Health Canada recommendations, defined as mercury blood concentrations between 20 μg/L (100 nmol/L) and 100 μg/L (500 nmol/L) [1]. If compared with a study conducted in 1992 by Dewailly *et al*. [5], mercury concentrations decreased for the same age group but still remain high (97.8 vs. 135.6 nmol/L in 1992) [31]. The comparison with mercury levels obtained in other populations is limited as we only included adults of 40 years and older and available data concerning adult exposure often include a wide age range.

Previous studies showing an impact of mercury on HRV have involved children exposed *in utero *[8,11,12] and adults suffering from FMD [10]. Changes in normal HRV were also detected in rats exposed to MeHg chloride [32]. In children exposed *in utero*, a negative influence of MeHg on HRV was detected in the Faroe Islands at 7 years of age [8] and this influence persisted when the same cohort was assessed at 14 years of age [11]. At 7 years of age, a decrease of HRV was found only in boys with mercury concentrations in the range from 1 to 10 μg/L (5–50 nmol/L) whereas at 14 years of age, only a slight decrease of HRV was observed in this range of concentrations. However, a decrease in sympathetic and parasympathetic activities was observed in the sample ranging from 0.9 to 351 μg/L (4.5–1755 nmol/L). Even if the results at 14 years of age are not similar to those obtained at 7 years of age, these results suggest an influence of MeHg on HRV in children exposed *in utero*. The authors suggest that changes observed in HRV could be the result of an influence of MeHg on the brainstem nuclei that regulates HRV. Furthermore, evidence indicates that the developing brain could be considerably more sensitive to the toxic effects of MeHg than the mature adult brain [33]. In a case-control study conducted by Oka *et al*. [10], they observed that individuals suffering from fetal Minamata disease (FMD) had lower NN and HF than controls. These results also support the hypothesis that the cardiac autonomic activity may be vulnerable to MeHg toxicity during the prenatal period. In this population, most of the cases recorded were probably exposed both prenatally and postnatally due to the long-term contamination of fish in the Minamata Bay but this could not be confirmed [33]. The hypothesis concerning the vulnerability of the cardiac autonomic activity to MeHg toxicity during the prenatal period is also supported by the results obtained by Murata *et al *[12]. In that study, MeHg exposure at parturition was associated with decreased vagal modulation of cardiac autonomic function while postnatal mercury exposure was not significantly related to the parasympathetic tone.

Although prenatal exposure to mercury has been suggested to influence the cardiac autonomic system, post-natal exposure could also have some influence on the ANS. In Faeroe Island, LF component was affected by mercury exposure at 7 years of age [11]. In our study, most of the HRV parameters decreased with blood mercury concentrations although only the association with SDANN was statistically significant. SDANN represents the long-term HRV and it correlates with SDNN. In this study, the association with SDNN approached the significance level and a similar trend was observed in the CVRR model. The fact that associations were in the same direction as those observed in previous studies [10-12], contributes to support the hypothesis that mercury exposure may influence HRV in adults.

Regarding BP, a previous study conducted in children exposed *in utero *has reported an increment in SBP [8] which is in accordance with the results from the present study. In Greenlanders adults, mercury in blood was associated with reduced DBP and increased PP [9]. Our results are partially in accordance with those observed in Greenlanders since PP increased with mercury concentrations in Inuit from Nunavik. Furthermore, our results are in accordance with results derived from hair's mercury measurement showing an association with increased SBP [20].

In the present study, we tried to minimize the confounding bias by assessing the influence of most of the HRV and BP risk factors described in the scientific literature. Also, the regression coefficients did not change significantly when analyses were conducted excluding individuals with systemic hypertension, myocardial infarction, stroke and those with other cardiovascular diseases. Furthermore, analysis was conducted by excluding individuals with antiarrhythmic or beta blocker medication and the HRV regression coefficients did not change significantly. In the BP analysis, exclusion of individuals with anti-hypertensive medication did not change the regression coefficients. Additionally to HRV and BP risk factors, we also evaluated the possible confounding effect of n-3 fatty acids which are of particular interest in Arctic populations. In Nunavik, levels of EPA and DHA increased linearly with mercury blood concentrations (r = 0.50, p < 0.0001; r = 0.60, p < 0.0001 respectively). n-3 fatty acids can positively modulate HRV [34-36] as DHA can slow the heart rate [37,38], prevent ventricular arrhythmias [39], and sudden death [40]. n-3 fatty acids could also have a protective effect on BP [41,42]. In BP analyses, we also included selenium since it is correlated with mercury levels (r = 0.63, p < 0.0001) and it could have a protective effect on BP [43]. In this study, selenium was a significant predictor in SBP model and close to the significance level for DBP and PP. Furthermore, selenium adjustment increased the mercury coefficients in all BP models. This aspect constitutes an important difference between our study and those previously published.

Among other confounding variables included in the final models, insulin sensitivity (measured as HOMA-IR) explained a significant proportion of the variance in most HRV models. It is known that the autonomic nervous system is impaired in patients with diabetes. HRV diminishes with higher blood glucose concentrations, possibly due to an increase in the sympathetic activity [14,44-46]. Other variables such as age and gender also explained a significant proportion of the variance in some models. It is known that HRV diminishes with age [47] and males are more at risk of reduced HRV than females [48,49]. Obesity was also associated with some of the HRV parameters. It has been observed that weight reduction improves HRV [23].

In the present study, most of the HRV regression coefficients did not reach the statistical significance although they were in the same direction as those previously reported. One of the limitations of this study is that data on HRV were collected as part of a general health survey and maybe statistical power was not high enough to detect significant associations with all HRV parameters. Also, the participation rate for ambulatory electrocardiograms was not high (34.2%) and this could bias the results if individuals excluded from the analyses have different profiles than those included in the study. However, the weighting method used allows us to minimize the bias due to non-response which consequently minimizes risk of selection bias. Among the main causes of refusal, 122 individuals (47%) did not sign a consent form. However, among other 144 exclusions [69 individuals with non-valid Holter (26%) and 75 (27%) excluded due to pacemarker, no time, unspecified, device not available, handicapped, etc)], no significant differences were observed in blood mercury levels compared to individuals included in final analysis. Thus, exclusion of these individuals was not linked to the exposure and consequently, the risk of selection bias is less probable. In addition, non-valid Holter were due to artefacts, bad connections, non-functional cassettes due to the Holter management and no individual was excluded due to poor heart function which may be reflected by extremely low HRV for example.

## Conclusion

The results obtained in the present study suggest a deleterious impact of mercury on BP and HRV in adults. Although only the association with SDANN was statistically significant, most of the regression coefficients were in the same direction as those observed in children. Taking into account that this is the first study regarding the effect of mercury on HRV in adults, the results obtained here should be confirmed in other populations.

## Abbreviations

HRV: heart rate variability; NN: average of all R-R intervals; SDNN: standard deviation of R-R intervals; SDANN: standard deviation of the average R-R intervals calculated over 5-minute periods; CVRR: coefficient of variation of RR intervals; rMSSD: square root of the mean squared differences of successive R-R intervals; pNN50: proportion of interval differences of successive R-R intervals > 50 ms; VLF: very low frequency; HF: high frequency; LF: low frequency; SBP: systolic blood pressure; DBP: diastolic blood pressure; PP: pulse pressure; WC: waist circumference; HOMA-IR: insulin sensitivity estimated from the HOMA model (Homeostasis model assessment); MeHg: methylmercury; ANS: autonomic nervous system; DHA: docosahexanoic acid; EPA: eicosapentanoic acid; LDL-cholesterol: low-density lipoprotein; HDL-cholesterol: high-density lipoprotein; ICP-MS: inductively coupled plasma mass spectrometry; FMD: fetal Minamata disease, CNS: central nervous system; ECG: electrocardiogram

## Competing interests

The authors declare that they have no competing interests.

## Authors' contributions

BV was involved in data analysis, interpretation of the results and in drafting the manuscript, ED has participated in the conception and design of the study, data collection and interpretation of the results, PP has participated in the conception and design of the study as well as in the interpretation of the results. All authors read and approved the final manuscript.
